# Dysregulated Intracellular Signaling in the Pathogenesis of Vitiligo: An Update on Emerging Therapeutic Strategies

**DOI:** 10.3390/biomedicines13092177

**Published:** 2025-09-05

**Authors:** Ramona Marrapodi, Alberto Marini, Barbara Bellei

**Affiliations:** Laboratory of Cutaneous Physiopathology, Integrated Center of Metabolomics Research, San Gallicano Dermatological Institute, IRCCS, 00144 Rome, Italy; ramona.marrapodi@ifo.it (R.M.); alberto.marini@ifo.it (A.M.)

**Keywords:** vitiligo, melanocytes, pigmentation, skin

## Abstract

Vitiligo is an acquired depigmentation disorder characterized by the selective destruction of melanocytes, resulting in the progressive loss of pigment in the skin and hair. This condition frequently leads to significant psychological distress. Its pathogenesis is complex and multifactorial, involving a combination of genetic susceptibility, metabolic derangement related to oxidative stress, defective melanocyte adhesion to the basal epidermis, and dysregulated innate and adaptive immune responses, ultimately converging in the targeted elimination of melanocytes. Despite the availability of several therapeutic modalities, current corrective options are often limited in efficacy and are associated with high relapse rates. There remains a pressing need for novel, safe, and more effective therapeutic strategies to improve patients’ quality of life. Growing evidence indicates that the immune system plays a pivotal role in vitiligo onset and progression, as most triggers converge on inflammatory and autoimmune pathways targeting melanocytes. However, immunosuppressive therapies alone have shown limited effectiveness in halting disease progression and achieving lasting repigmentation. Targeting only immunological processes without addressing the underlying triggers of their activation likely represents a significant limitation in restoring pigmentation. In contrast, interventions aimed at upstream events may help prevent the initiation of the immune response. Consequently, combinatorial therapeutic approaches that target multiple pathogenic pathways and incorporate diverse pharmacological agents are being explored to improve clinical outcomes. This review aims to re-evaluate the intrinsic cellular abnormalities and associated dysregulated signaling pathways in vitiligo, with the goal of identifying novel, effective, nonimmunological treatment strategies.

## 1. Introduction

Epidermal melanocytes are specialized pigment-producing cells derived from the neural crest, located in the basal layer of the epidermis, where they play a central role in skin pigmentation and photoprotection. Melanocytes synthesize melanin within specialized organelles called melanosomes through a multi-step enzymatic process that begins with the oxidation of tyrosine, catalyzed by tyrosinase. The two primary forms of melanin produced are eumelanin (brown/black) and pheomelanin (red/yellow), which vary depending on genetic and environmental factors. Once produced, melanin is transferred to surrounding keratinocytes, where it contributes to skin pigmentation and protects against ultraviolet (UV) radiation [1]. Melanocyte activity is tightly regulated by intrinsic factors, such as Microphthalmia-associated transcription factor (MITF), and extrinsic signals from the surrounding microenvironment, including UV radiation and cytokines [2,3]. In addition to their role in pigmentation, melanocytes contribute to local immune responses and redox balance, underscoring their multifaceted function in skin homeostasis [4]. Accordingly, alterations in melanocyte function or survival are associated with various pigmentary disorders and may also reflect broader dysfunctions unrelated to pigmentation alone.

During life, the phenotype of cutaneous pigmentation changes within the range of non-pathological conditions, as a function of age, the environmental exposome, trauma, diet, and drug use. However, occasionally in life, individuals may develop skin pigmentation disorders, which can arise unexpectedly and impact both appearance and quality of life. Pigmentary disorders are generally classified as either hypopigmentation or hyperpigmentation and can result from genetic mutations or acquired conditions. Acquired pigmentary abnormalities may present as localized lesions or diffuse skin involvement and include conditions such as vitiligo, melasma, and post-inflammatory hypopigmentation [5]. Among these, vitiligo represents the most common form of acquired depigmentation.

Vitiligo is the most common depigmenting disorder, affecting 0.1–2% of the population worldwide without differences related to skin phototype [6]. Vitiligo is characterized by visible white spots and occurs when epidermal melanocytes, which produce the pigment melanin, die or cease to be functionally active [7]. In vitiligo patients, pigmentary abnormalities sometimes involve hair, eyelashes or eyebrows, and eyes [8]. Vitiligo is classified as a non-segmental (NSV, characterized by symmetrical depigmentation of the skin that affects both sides of the body), segmental (SV, affects only one side of the body or a specific area), or mixed form (simultaneous presence of NSV and SV) [9]. Even if vitiligo is confined to the skin, and is not contagious or life threatening, it can lead to relevant psychological stress and frequently have a significant impact on daily life [10].

**Figure 1:**
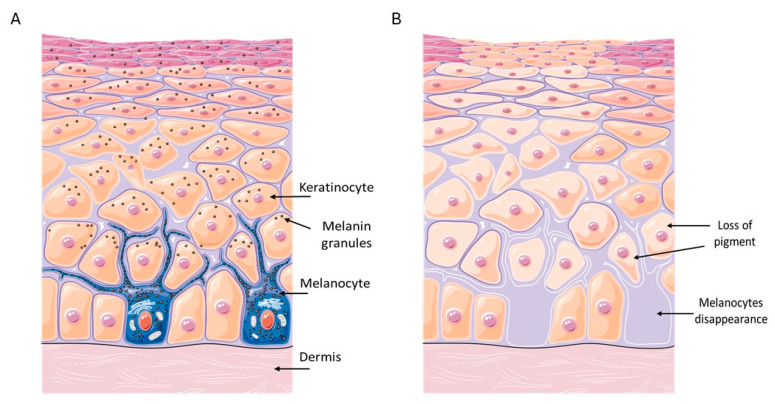
Schematic representation of normal skin (**A**) and vitiligo-depigmented skin lacking melanocytes and melanin (**B**).

The pathogenesis of vitiligo is not fully understood, but it is thought to be intricate, involving genetic components, metabolic factors linked to cellular oxidative stress, defects in melanocyte adhesion to the epidermis, and immunity (innate and adaptive), which culminate in selective melanocyte destruction (Figure 1) [11].

However, none of these mechanisms alone fully explain the process of melanocyte disappearance, indicating that the disease is multifactorial. Correspondingly, treatment of vitiligo is challenging, with great variability in terms of disease extent, distribution, and activity [12]. As a result, there is a significant demand for treatment among patients. Progress in understanding the underlying mechanisms of vitiligo facilitates the development of targeted therapies. Emerging treatments, including biologics designed to target cytokines, are emerging as effective therapeutic options [13]. One example is ruxolitinib, a topical janus kinase 1/2 (JAK1/JAK2) inhibitor very recently approved in both the US and Europe for adult and pediatric NSV patient management [14]. It is the first pharmacological treatment approved for vitiligo. JAK activity is positively correlated with increased CD8^+^T release of inflammatory mediators of the JAK1–signal transducer and activator of transcription 1 (STAT1)–interferon γ (IFNγ) axis [15]. However, the engagement of the immune system may represent the final phase of a complex and protracted process. From the perspective of pathogenic mechanisms, considering melanocyte clearance as the “tip of the iceberg,” therapeutic interventions targeting the initiating events may prove more effective than those merely suppressing the immune system. The chronic and often relapsing nature of vitiligo, frequently persisting for a lifetime, reframes the approach to therapeutic treatment. Furthermore, the risks associated with prolonged immune suppression must be carefully considered. To develop innovative therapeutic strategies, it is essential to further elucidate the underlying pathogenic mechanisms.

This review presents and analyzes both experimental and clinical data supporting the therapeutic targeting of intracellular pathways in melanocytes. Emphasis is placed on early initiating factors in vitiligo pathogenesis, such as oxidative stress and metabolic dysfunction, which may offer strategic entry points for intervention. The discussion also explores potential future therapeutic developments aimed at restoring melanocyte function and improving disease outcomes.

## 2. Targeting Vitiligo-Impaired Redox Defense Network

Reactive oxygen species (ROS) are free radicals derived from molecular oxygen, including superoxide anion (O_2_•^−^), hydroxyl radical (HO•), and non-radical molecules such as hydrogen peroxide (H_2_O_2_) [16]. They are derived from both intracellular (e.g., mitochondria, peroxisomes, endoplasmic reticulum) and extracellular (e.g., ultraviolet radiation, air pollution, alcohol, tobacco smoke, heavy metals, transition metals, industrial solvents, pesticides) sources [17,18,19]. Physiological ROS production is strictly regulated because ROS are not simply metabolic by-products but at moderate levels can also function as secondary messengers modulating various harmful cellular processes [20]. However, excessive ROS production or inadequate detoxification leads to loss of oxidative equilibrium, causing cellular damage and contributing to the development and progression of various diseases [21,22,23]. The main molecular targets of free radicals include (i) nucleic acids with fragmentation of DNA filament, point mutation, and alteration of gene expression; (ii) structural and functional change in proteins; (iii) lipid peroxidation that compromises membrane integrity and function; (iv) carbohydrates, since glycosylation machinery promptly responds to oxidative stress [24]. There is robust evidence that ROS contribute to a wide range of inflammatory and autoimmune diseases including diabetes, lupus, and rheumatoid arthritis [21]. Compromised oxidative equilibrium is also extensively documented in the chronological and premature appearance of a skin aging phenotype [25,26]. Furthermore, ROS have a multifaceted impact on the onset and development of several types of cancer, including melanoma and nonmelanoma skin cancers, due to the impact on p53, kelch-like ECH-associated protein 1, nuclear factor erythroid 2-related factor 2 (Keap1-Nrf2) complex, wingless (Wnt)/β-catenin, retinoblastoma transcriptional corepressor 1 (RB1), and mitogen-activated protein kinase (MAPK) pathways [27].

### 2.1. Clinical and Experimental Evidence Supporting Oxidative Stress Targeting in Vitiligo

**Figure 2:**
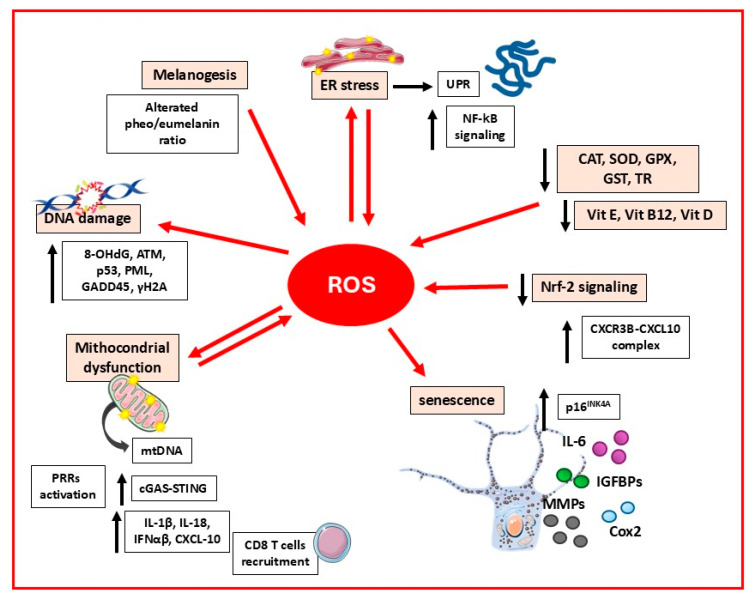
Summary of the damage and associated pathways due to oxidative stress in vitiligo. The accumulation of reactive oxygen species (ROS) in melanocytes results in extensive intracellular damage. Mitochondrial dysfunction, DNA damage, endoplasmic reticulum (ER) stress, and impairment of Nrf2-mediated antioxidant signaling collectively contribute to redox imbalance. This oxidative environment promotes melanocyte senescence and predisposes cells to apoptosis.

The central role of oxidative stress in the onset and progression of vitiligo is amply documented (Figure 2) [6,28,29].

Loss of oxidative equilibrium in vitiligo skin is demonstrated by extensive accumulations of H_2_O_2_ (in the millimolar range) in the lesional and nonlesional epidermis and serum of vitiligo patients [30,31,32,33]. Parallel analysis of peripheral blood mononuclear cells (PBMCs) and the epidermal antioxidant network in vitiligo patients showed a tight correlation evidencing the intrinsic nature of redox disequilibrium [34]. The high level of ROS in vitiligo is a consequence of abnormal production and of reduced activity of antioxidant enzymes comprising catalase (CAT), superoxide dismutase (SOD), glutathione peroxidase (GPX), glutathione S-transferase (GST), thioredoxin reductase (TR), and methionine sulfoxide reductases (Msrs) [35,36]. Also, several antioxidant small molecules such as vitamin E, vitamin B12, vitamin D, and coenzyme Q have been found to be altered in vitiligo patients [37].

The persistence of oxidative disequilibrium in vitiligo melanocytes leads to sustained activation of antioxidant enzyme gene expression at the mRNA level [38]. Despite the increased mRNA expression of these enzymes, the protein levels of CAT and superoxide dismutase 2 (SOD2) remain low, indicating an inherently higher turnover of detoxifying enzymes that are not dependent on external stressors. Moreover, this extreme situation renders vitiligo melanocytes not fully competent to counteract oxidative injury, as evidenced by the incapacity to stimulate the production of detoxification enzymes after treatment with tert-butyl-hydroperoxide, a membrane-permeant oxidant [38]. This phenomenon, characterized by elevated expression levels of antioxidant proteins, coupled with the reduced functional activity might reflect an adaptive response where the increased enzyme expression seeks to compensate for the reduced enzymatic capacity, and it is commonly observed in conditions of prolonged oxidative stress, such as in Alzheimer’s disease [39]. This likely reflects a heightened demand for enzyme expression due to the reduced activity, which may result directly from chronic oxidative damage. Alteration of several oxidative stress-linked signaling pathways has been identified as a possible causative reason for vitiligo, indicating a potential therapeutic strategy to modulate ROS levels, prevent cell damage, and restore melanocyte function in vitiligo skin. Compared to controls, vitiligo melanocytes under H_2_O_2_-induced oxidative injury retain reduced Nrf2 nuclear translocation and transcriptional activity. Indeed, overexpression of Nrf2 attenuates H_2_O_2_-induced cytotoxicity in PIG3V, an immortalized vitiligo cell line [40]. Recently, Le et al. demonstrated that impaired Nrf2 signaling under the pressure of oxidative stress in vitiligo melanocytes determines a C-X-C motif chemokine receptor 3 isoform B-C-X-C motif chemokine ligand 10 (CXCR3B-CXCL10)-dependent apoptosis, directly linking oxidative stress to inflammation [41]. This data is relevant since CXCR3-deficient T cells exhibit reduced skin-homing capacity and a diminished ability to induce depigmentation [42]. Moreover, CXCR3 may contribute to vitiligo relapses, as depigmentation frequently recurs at previously affected sites, suggesting the role of resident memory T cells (TRMs). Functional CD8+ TRMs have been identified in both active and stable vitiligo, with CXCR3 expression found in most of these cells [43]. Moreover, the number of CXCR3B+ melanocytes is increased in active vitiligo skin compared to control samples and stable/repigmented vitiligo [44]. CXCR3 has long been viewed as a promising therapeutic target in vitiligo, given its key role in disease progression. CXCR3-blocking antibodies have shown efficacy in preventing and reversing depigmentation in mouse models [45], and various inhibitors are currently under development as potential treatments [42,46].

#### 2.1.1. Melanocytes Are Intrinsically Prone to Lose Oxidative Equilibrium

In addition to extrinsic sources, ROS production in melanocytes can also arise from a lineage-specific intrinsic propensity associated with the melanogenic biosynthetic pathway, which consumes large amounts of adenosine triphosphate (ATP) and requires lipid resources for the assembly of melanosomes, lysosome-derived organelles dedicated to the synthesis, storage, and transport of melanin [47], resulting in free radical species production. Furthermore, even if confined to melanosomes, abundant intracellular melanin might be toxic. Based on the relative eumelanin and pheomelanin contents, the intracellular milieu might present pro-oxidant features since reddish/brownish pheomelanin has spontaneous and UV-induced pro-oxidant properties, whereas brown/black eumelanin protects against injuries [48]. An interesting report by Parsad’s group highlighted the prevalence of pheomelanin as the residual pigment in depigmented lesions, whereas PUVA-induced repigmentation, which typically follows a perifollicular pattern originating from hair follicles, consists predominantly of eumelanin [49]. In line with the idea that the pheo/eumelanin ratio could be perturbed during vitiligo evolution, Yonemoto and collaborators demonstrated that chemically induced depigmentation specifically compromises eumelanin synthesis [50]. Studies concerning vitiligo prevalence demonstrated a higher value in type III (light-brown skin) and type IV (moderately brown skin) phototypes [51]. Confirming the general lower antioxidant defense in vitiligo subjects, Briganti et al. reported that the direct correlation between skin phototype, and constitutive antioxidant status is maintained in vitiligo skin [34]. More importantly, the trend for an endogenous antioxidant system seems to be not simply related to the presence of melanin but a proper individual characteristic, since the antioxidant profile of vitiligo lesional skin still correlated with those of corresponding PBMCs [34]. Physiologically, the presence of melanin in cultured primary human melanocytes is associated with high catalase expression and activity [52] but with a low level of glutathione (GSH) [53]. Moreover, the increase in CAT expression and its redistribution into melanosomes by α-melanocyte-stimulating hormone (α-MSH), the major hormone controlling melanogenesis, highlights the necessity of protection against oxidative stress in the case of intense pigment production [54]. Furthermore, increased levels of intracellular melanin positively correlate with the expression of the DNA damage marker γ-H2A histone family member X H2AX (γH2AX) [55]. As a result, melanocytes are more prone to losing oxidative equilibrium than most other skin cell types [56].

#### 2.1.2. Oxidative Stress-Related Intracellular Damage

At high concentration, ROS destroy melanocyte biology in all aspects, including damage to DNA, lipids, proteins, and metabolites both structurally and functionally. Advanced oxidation protein products (AOPPs), peroxidated lipids, propanediol, and 8-hydroxy-2-deoxyguanosine (8-OHdG) are markers of vitiligo progression [57,58]. Oxidative DNA damage is present in the skin of vitiligo patients, where 8-OHdG accumulates compared to healthy individuals [59]. Salem et al. observed overexpression and activation of the DNA lesion sensor ataxia telangiectasia-mutated (ATM) kinase, which in turn activates p53 by phosphorylation [59]. Also, our group demonstrated chronic activation of p53 and its target genes promyelocytic leukemia (PML) and growth arrest and DNA damage-inducible protein alpha (GADD45) in normally pigmented biopsies of vitiligo patients, highlighting the intrinsic nature of vitiligo-associated damage and its independence from pigment loss [38]. Compared to healthy controls, patients with active vitiligo also show increased levels of 8-OHdG in mononuclear leukocytes, which agrees with systemic ROS-mediated DNA damage [60,61]. However, the robust activation of DNA repair mechanisms via 8-oxoguanine DNA glycosylase (hOgg1), apurinic/apyrimidinic endodeoxyribonuclease 1 (APE1), and DNA polymerase β explains the absence of heightened solar sensitivity, photodamage, skin aging, or an increased incidence of sun-induced nonmelanoma skin cancer in vitiligo patients [62]. In contrast, vitiligo melanocytes exhibit a tendency to accumulate somatic variants in mitochondrial DNA (mtDNA) [63]. Consistently with the limited repair mechanisms for mtDNA compared to genomic DNA, unrepaired mtDNA is released into the cytoplasm of vitiligo cells, leading to the activation of the cyclic GMP-AMP synthase–stimulator of interferon genes (cGAS-STING) pathway, which subsequently triggers immune response [63,64]. Notably, Schallreuter et al. proposed a role of estrogens’ oxidative metabolism in contributing to DNA damage in peripheral blood lymphocytes of vitiligo patients by inducing ROS production. These data may explain why estrogen-associated conditions such as pregnancy, postpartum disorders, hormone replacement therapy, and use of oral contraceptives are correlated with the onset or rapid progression of the disease [65].

Additionally, ROS-induced oxidative stress progressively triggers abnormal organelle functions, disrupts metabolic pathways, and weakens the protective mechanisms against further oxidative agents [66]. At low concentration levels, as part of the DNA damage response, H_2_O_2_ induces premature melanocyte senescence through a p53-independent p21 pathway [67]. In senescent melanocytes, dendrite formation and intercellular adhesion ability are compromised, thereby impairing the melanosome transfer function, which may cause skin depigmentation. On the other hand, N-acetylcysteine (NAC) supplementation counteracts the acquisition of the senescent phenotype, and the impaired melanosome transfer induced by H_2_O_2_ [67]. Dedifferentiation, at least at an early stage of disease, might explain depigmentation, since several studies demonstrated the presence of functional compromised melanocytes in depigmented patches [68,69]. Melanocytes surrounding depigmented vitiligo skin present high levels of p16^INK4A^ expression independently from chronological age, reinforcing the idea that the acquisition of senescent-prone features precedes melanocyte disappearance [70]. Senescence has been demonstrated to be part of the degenerative process of vitiligo pathogenesis, as demonstrated by the anomalous expression of a multitude of proteins associated with the senescence-associated secretory phenotype (SASP) including interleukin-6 (IL-6), cyclooxygenase (Cox-2), extracellular matrix metalloproteinases (MMPs), and insulin-like growth factor-binding proteins (IGFBPs) [38]. It is reasonable that as part of premature aging of vitiligo skin, immature committed cells or pluripotent stem precursors lose the capacity to replace damaged/missing melanocytes. The acquisition of early senescence-associated features by skin cells links vitiligo to a panel of degenerative diseases such as cardiovascular pathologies [71], neurodegeneration [72], and ocular diseases [73], suggesting some possible shared therapeutic approaches. In addition, this implies the possible use of natural or synthetic senolytic compounds in vitiligo therapy. However, senescence of melanocytes and of their microenvironment has recently also been reported in hyperpigmentation disorders such as melasma and age-spots (reviewed in [74]), complexifying data interpretation. A divergent aspect of hypopigmentation- and hyperpigmentation-associated senescence emerges in comparing growth factor production, since hypersecretion of growth factors has been frequently reported as a peculiar feature of fibroblasts underlying hyperpigmented spots [75,76], whereas vitiligo skin is relatively poor in growth factors produced by fibroblasts and keratinocytes [77,78]. Sub-physiological stimulation of melanocytes by neighboring cells might explain the infrequent spontaneous repigmentation of vitiligo skin. Furthermore, since senescent cells are ordinarily recognized and cleared by the immune system [79], the persistence of this condition may promote the production of autoantigens, which could drive autoimmunity and contribute to the sustained depigmentation observed in vitiligo. In melanocytes, massive accumulation of ROS changes mitochondria structure and morphology, leading to cell death [80], a mechanism that could in part explain pigment cell disappearance. In cultured vitiligo melanocytes, augmented ROS are associated with consistent metabolic impairment [81], discussed separately in this review. Moreover, in vitro, melanocytes exposed to mitochondrial-driven oxidative stress accumulate misplaced cytoplasmic mtDNA [82]. The relevance of misplaced mtDNA consists in its capacity to function as an endogenous trigger of inflammation [83]. In vitiligo melanocytes, high frequency of somatic mtDNA variants correlates with lower ROS scavenging capacity, augmented release in the culture supernatants of interleukin 1-β (IL1-β), IL-18, interferon α and β (IFNα/β), C-X-C motif chemokine ligand 9 (CXCL9), and C-X-C motif chemokine ligand 10 (CXCL10), resulting in strong PBMC recruitment [63]. Recent studies have detected elevated levels of mtDNA in both the serum and depigmented lesions of vitiligo patients [84]. In the same study, the administration of exogenous mtDNA in a murine model significantly accelerated the progression of vitiligo, as demonstrated by pronounced depigmentation of the tail, accompanied by intense CD8+ T cell infiltration and a reduced proportion of regulatory T cells (Tregs) [84].

A relevant consequence of oxidative stress in vitiligo is the accretion of unfolded/misfolded proteins in the endoplasmic reticulum (ER), which activates the unfolded protein response (UPR) to restore cellular homeostasis [85,86]. Notably, ER stress is an important player that participates in melanocytic cell death in vitiligo patients by increasing melanocytes’ immunogenicity [87]. Since the ER is the major site of membrane lipid synthesis and recycling, impairment of ER function might impact the membrane composition of the cell membrane and other organelles as well as vesicular traffic [88]. The ER is the site of gluconeogenesis, and its dynamic shaping contributes to autophagosome and peroxisome biogenesis, an important step for the degradation of dysfunctional organelles or a portion of them [89].

The amino acid metabolite homocysteine, whose levels are highly increased in vitiligo patients at progressive stages, has been demonstrated to induce the death of melanocytes associated with vitiligo by triggering ER stress and activating the UPR in a ROS-dependent manner [90]. Emerging evidence highlights the crucial role of the UPR in immune regulation. UPR contributes significantly to stimulatory effects on the nuclear factor kappa-light-chain-enhancer of activated B cells (NF-κB) pathway [91]. Furthermore, impaired UPR has been associated with the development of autoimmunity in various disorders including inflammatory bowel disease, arthritis, neurodegenerative diseases, systemic lupus erythematosus (SLE), and diabetes mellitus [92,93]. Thus, it is plausible that ER stress participates in the initial development of vitiligo autoimmunity, suggesting that UPR-modulating strategies might be of interest for vitiligo patients.

### 2.2. Clinical Trials Supporting the Relevance of Impaired Redox Equilibrium in Vitiligo

Supplementation with antioxidants has gained attention in vitiligo treatment. These include enzymes, and a series of small molecules like vitamins and minerals that help to protect cells from damage caused by free radicals. All these strategies have been proposed for topical or systemic treatment of vitiligo, yielding promising results [94]. Authorized clinical trials are reported in Table 1.

#### 2.2.1. Clinical Trials Involving Vitamin Supplementation

Among strategies, vitamin D, vitamin C, vitamin E, lipoic acid, α-tocopherol, and selenium supplementation [95] provides beneficial effects in counteracting progression and, in some cases, enhancing intervention outcomes. However, evidence for their efficacy as primary therapies is limited [96]. According to a study by Finamor et al., patients receiving a high dose of vitamin D3 supplementation for six months achieved clinical improvement, suggesting that vitamin D could help to reduce disease progression [96]. In support of the use of vitamin integration, lower vitamin D levels have been observed in vitiligo patients compared to age- and gender-matched healthy subjects, whereas contrasting results have been reported for blood concentration of vitamins C and E [97,98]. Of note, hypovitaminosis D increases the risk of autoimmunity onset. However, dysfunction of vitamin D activity was related to an elevated frequency of dysfunctional polymorphic vitamin D receptor variants in vitiligo patients [99]. Vitamin C (L-ascorbic acid) is a hydrophilic antioxidant that supports melanocyte function. Vitamin C supplementation ameliorates the lipid and inflammatory profile of vitiligo patients’ serum, and a randomized clinical trial provided evidence for oxidative stress and pigmentation improvement [100]. Vitamin C has been proposed to treat both depigmented and hyperpigmented spots [101]. The inhibitory activity of vitamin C on tyrosinase (TYR), the rate-limiting enzyme for melanin synthesis, explains the skin-lightening properties of vitamin C and cast doubt on its use in vitiligo therapy. Vitamin E is a lipophilic free radical scavenger that protects cell membranes from damage caused by free radicals. NAC, acting as a precursor to glutathione and as a direct free radical scavenger, helps to reduce oxidative damage [102]. The addition of vitamin B9 and B12, both wanting in patients, in the diet for a long period demonstrated some benefits in terms of repigmentation [103].

#### 2.2.2. Clinical Trials Involving Antioxidant Supplementation

Extract of Polypodium Leucotomos (PL), a tropical fern, with free radical quenching capacity potentiates the narrow-band (NB) UVB repigmentation capacity [104]. However, data demonstrating a marked immunomodulatory and photoprotective effect of PL, reflected by a diffuse use in other skin conditions such as psoriasis and atopic dermatitis, indicates a more complex scenario [105,106].

Replacement of nonfunctional CAT with cream containing pseudocatalase (a compound designed to mimic the activity of the natural enzyme catalase) represents an additional therapeutic antioxidant strategy. Topical application of pseudocatalase (PC-KUS) combined with its activation by NB-UVB radiation or natural sun has shown contrasting data, with some studies reporting benefits and others showing limited or no significant therapeutic effects [107,108,109,110,111,112]. Nevertheless, information regarding pseudocatalase’s safety profile and possible adverse effects is limited.

Lastly, trace elements such as copper and selenium, even if barely present in humans, are important for protection of cells from free radicals, and their integration has been proposed by some clinical studies, even if change in the serum concentration of these elements in patients with vitiligo is still debated [113]. Researchers noted a decline in serum levels of zinc and copper in vitiligo patients compared to healthy controls, whereas results regarding selenium are highly heterogeneous [37]. Selenium plays a crucial role in the activity of the GPX family of enzymes, indicating possible beneficial effects of its dietary integration.

Although antioxidant therapy shows promise in treating vitiligo, it is often considered as part of a broader treatment approach, alongside other therapies such as phototherapy, immunosuppressants, and melanocyte transplantation.

## 3. Proliferation, Cell Death, and Senescence

Accelerated cell death of mature melanocytes, faint mobilization, proliferation, and differentiation of melanocyte stem cell populations, and the occurrence of premature senescence have been all implicated in vitiligo. Designing pharmacological compounds that can specifically target these activities has been widely proposed to achieve complete durable pigment recovery in vitiligo patients.

### 3.1. Stimulating Precursor Melanocyte Mobilization, Proliferation, and Differentiation

Epidermal melanocytes are normally slowly dividing cells, even if wounding and UV exposure might intermittently stimulate melanocyte lineage mitotic activity. Repigmentation of vitiligo requires an increase in the number and migration of melanocytes into the depigmented epidermis. This could be realized by stimulating resident melanocyte precursors localized within the niche in the bulge of the hair follicle [114,115] and in the interfollicular melanocytes that have already migrated from the hair follicle bulge and present variable differentiation status [116]. The phototherapy therapeutic option (NB-UVB; psoralen plus ultraviolet A, PUVA; excimer laser lamp) is the first-line treatment. Phototherapy acts as an immunosuppressor and coordinates melanocyte precursor stimulation [117]. This results from the direct stimulation of stem cells in the hair bulge and the targeting of the microenvironment, as melanocyte proliferation is physiologically influenced by interactions with surrounding cells, particularly keratinocytes and fibroblasts [118]. Also, sebocytes, epithelial cells specialized in sebum production, participate in the crosstalk between skin cells that regulate pigmentation, influencing skin color in areas of the body rich in sebaceous glands [119]. Consequently, complete cutaneous homeostasis is important for pigmentation recovery.

Reduction in fibroblast-derived growth factors that support melanocytes’ normal activities may play a role in the development of vitiligo. In vitiligo lesions, the levels of basic fibroblast growth factor (bFGF), stem cell factor (SCF), endothelin-1 (ET-1), granulocyte–macrophage colony-stimulating factor (GM-CSF), and α-MSH, which are released by keratinocytes and fibroblasts, are lower in the skin and blood of patients compared to healthy controls [120,121]. Of note, augmented melanocortin 1 receptor (MC1R) expression in the nonlesional skin of vitiligo patients compared with controls may represent an attempt to restore normal pigmentation when the ligand is barely produced [122,123]. Direct skin delivery of α-MSH enhances tofacitinib- and NB-UVB-induced repigmentation in vitiligo [124,125], and a lower amount of circulating α-MSH has been associated with poor outcomes in the case of melanocyte transplantation [126], suggesting that its supplementation (active synthetic substance named Afamelanotide) might be useful as a combined therapy strategy. Clinical trials combining Afamelanotide with NBUVB phototherapy demonstrated statistically significantly superior and faster repigmentation compared with phototherapy alone [127] (see Table 1). The interest concerning α-MSH analog peptides is not restricted to promelanogenic activity, because several studies have demonstrated antioxidant and anti-inflammatory properties [128]. Lastly, the topical use of bFGF-related decapeptide solution in combination with the immunosuppressive drug Tacrolimus (a calcineurin inhibitor) has shown improved skin repigmentation compared to Tacrolimus-alone therapy [129]. Insulin-like growth factor 1 (IGF-1) plays a crucial role in melanocyte biology due to its involvement in proliferation, melanogenesis, and protection against ROS in melanocytes. Recently, our group demonstrated insulin (Ins) and IGF-1 resistance at the cellular level in melanocytes, keratinocytes, and fibroblasts, highlighting that the entire skin is implicated in metabolic dysfunction, which may contribute to the pathogenic mechanism [130]. Further compounding this issue, El-Komy and colleagues reported significantly lower levels of IGF-1 in the serum and skin of vitiligo patients [131]. Additionally, IGF-1 activity is modulated by several IGF-binding proteins that regulate its binding to receptors. The expression of IGFBP3, 5, and 7 is notably higher in vitiligo melanocytes [38] and fibroblasts [74]. Given the elevated concentrations of growth factors such as vascular endothelial growth factor (VEGF), epidermal growth factor (EGF), IGF, platelet-derived growth factor (PDGF), fibroblast growth factor (FGF), and transforming growth factor β (TGFβ), autologous platelet-rich plasma (PRP) has been investigated in several clinical trials, combining this regenerative approach with autologous graft protocols and phototherapy [132]. Another promising autologous source of growth factors such as SCF, α-MSH, GM-CSF, bFGF, keratinocyte growth factor (KGF), and nerve growth factor (NGF) is the extracellular fraction of lipoaspirates, which contains a rich adipocyte secretome. In vitro studies have shown promising results for vitiligo dermal and epidermal cells [133].

### 3.2. Targeting Melanocyte Cell Death

In certain circumstances, several cellular insults reported in vitiligo cells might converge on melanocyte death. In line with this principle, persistent melanocyte damage might accelerate the turnover of this type of cell, leading to premature melanocyte stem cell exhaustion and limited regeneration capacity. Thus, protecting pigment cells from death might be important to stabilize disease progression and preserve repigmentation capacity.

**Figure 3:**
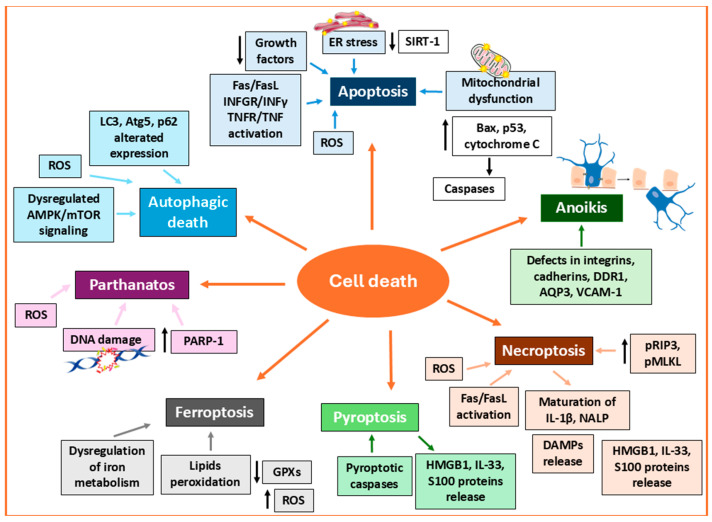
Representation of different mechanisms of cell death involved in the disappearance of melanocytes in vitiligo skin. Multiple regulated cell death pathways are activated in melanocytes from vitiligo patients, as indicated by changes in specific markers and signaling cascades. Each pathway contributes to melanocyte loss and may trigger the release of damage-associated molecular patterns (DAMPs), thereby promoting immune cell recruitment and perpetuating autoimmunity.

Cell death may occur through multiple types of well-regulated overlapping, but not identical, modalities including apoptosis, necroptosis, pyroptosis, and autophagy, all documented in vitiligo (Figure 3).

#### 3.2.1. Apoptosis

Apoptosis is a tightly regulated type of cell death mechanism characterized by an ordered sequence of events involving caspases, which lead to the cleavage of various proteins with distinct functions [134]. Since apoptosis, even when triggered by extrinsic insults, proceeds through a coordinated sequence of intracellular events, it is frequently analogized to a form of cell suicide. Due to the rapidity of its evolution and the limited inflammation derived, apoptosis is considered minimally immunogenic [6]. The intrinsic pathway of apoptosis is triggered by intracellular stimuli such as DNA damage, cell stress, mitochondrial dysfunction, and excessive intracellular ROS concentration [135]. The intrinsic signaling pathways that initiate apoptosis mostly involve mitochondrial events converging in the activation of the B-cell lymphoma protein-2 (Bcl-2) family of proteins (pro-apoptotic and anti-apoptotic) and p53, and the release of cytochrome c via alteration of mitochondrial membrane permeability [136]. Thus, it is not surprising that perturbation of mitochondria homeostasis easily opens the way to apoptosis. The changes in mitochondria in vitiligo were first identified in PBMCs from patients in active stages, which were associated with increased ROS production, a significant decrease in mitochondrial membrane potential, and inadequate ATP assembly [137]. Mitochondrial morphology and function changes have been observed in melanocytes, keratinocytes, and fibroblasts in lesional, perilesional, and nonlesional skin of individuals with vitiligo [138]. An altered ratio of Bcl-2/BCL2-associated X protein (Bax) (anti-apoptotic and pro-apoptotic, respectively) was detected in perilesional vitiligo melanocytes as compared to the controls [139]. H_2_O_2_-induced demethylation of the promoter region in transient receptor potential cation channel subfamily M member 2 (TRPM2) causes mitochondria-dependent melanocyte apoptosis via excessive calcium influx into the cytoplasm and loss of intracellular and mitochondrial calcium homeostasis. Of note, the elevated level of TRPM2 expression in vitiligo skin suggests this mechanism as a possible facilitator of vitiligo melanocyte loss [140].

An interesting link between apoptosis and impaired mitochondrial dynamics in vitiligo melanocytes emerged in a study proving excessive optic atrophy 1 (OPA1)-dependent inner membrane fusion, loss of normal cristae shape and resultant mitochondrial fragmentation associated with increased Bax expression, cytochrome c release from mitochondria to the cytoplasm, and activation of caspase 9, which ultimately triggers the intrinsic apoptotic program [141]. It has been reported that, in the presence of oxidative stress, the ER protein calreticulin is redistributed from the ER lumen to the cell membrane and that it is associated with apoptosis induction [142]. Acute ER stress is causative of apoptotic death in vitiligo, a process that seems to be facilitated in vitiligo melanocytes due to the low level of NAD-dependent protein deacetylase sirtuin-1 (Sirt1) expression [143]. By modulating Akt serine/threonine kinase (Akt) and downstream MAPK signaling, Sirt1 attenuates the expression of pro-apoptotic proteins protecting perilesional keratinocytes from stress-induced apoptosis [144]. These observations support the potential of SIRT1 activation as a protective strategy to preserve perilesional keratinocyte integrity in vitiligo. Alongside upstream apoptosis inducers, an imbalance between anti-apoptotic (Bcl-2) and pro-apoptotic (Bax) proteins in perilesional melanocytes suggests a dysregulated intrinsic apoptotic process [139]. The impact of apoptosis differs a lot based on the cell-specific proliferation dynamics. In keratinocytes, cell death is easily compensated for by the intense proliferation rate. Differently from keratinocytes, melanocytes, having a limited proliferative capacity, prevent apoptosis occurrence by Bcl-2 expression. Consistently, Bcl-2-deficient mice exhibit premature hair graying that progresses to ample hypopigmentation because of melanocyte apoptosis [115,145]. In melanocytes, apoptosis might also be a consequence of growth factor withdrawal [146]. As discussed above, the levels of bFGF, SCF, ET-1, α-MSH, and GM-CSF are lower in vitiligo lesions compared to healthy controls [120,121].

Several studies proposed that deficient adhesion contributes to the disappearance of melanocytes in vitiligo through anoikis, a form of apoptosis triggered by the loss of anchorage to the extracellular matrix and neighboring cells. Adhesion defects involving β1-integrin, cadherins, laminin, aquaporin-3 (AQP3), discoidin domain receptor tyrosine kinase 1 (DDR1), and vascular cell adhesion molecule-1 (VCAM-1) have been documented both in vivo and in vitro [147,148,149,150,151]. This condition results in a greater susceptibility to anoikis in vitiligo melanocytes due to cells’ defective anchorage to the surrounding extracellular matrix [139]. Melanocyte detachment disrupts fibronectin adhesion-mediated apoptosis suppression, and its shift toward migrating to the upper layer of the epidermis reduces melanocyte survival. In line with these findings, in patients with unstable vitiligo, melanocytes are poorly attached to type IV collagen, showing activation of caspase 3 and increased staining of the apoptotic marker Annexin V, while stable vitiligo and control melanocytes firmly adhere to type IV collagen [139,152]. In vivo mechanical stress such as friction or trauma of perilesional NS vitiligo skin causes the detachment of living melanocytes from the basal layer, leading to trans-epidermal migration and the eventual loss of the detached pigment cells, a process defined “melanocytorrhagy” [152]. The importance of proper cell–cell interactions, particularly those mediated by E-cadherin between keratinocytes and melanocytes, has been highlighted in a study by Wagner and colleagues. The authors demonstrated a discontinuous pattern of E-cadherin staining on melanocyte membranes in vitiligo patients, observed well before the onset of clinical manifestations [153].

In addition to their intrinsic propensity toward self-destruction, vitiligo melanocytes exhibit a markedly reduced ability to defend themselves against T cell-mediated attacks by engaging immunotolerance mechanisms. Unlike other skin cell populations, such as keratinocytes and fibroblasts, vitiligo melanocytes fail to upregulate the immunosuppressive membrane molecule programmed death-ligand 1 (PD-L1) in response to autoreactive T cells, thereby increasing their susceptibility to apoptotic insults [154]. The extrinsic apoptotic pathway involves transmembrane receptor-mediated interactions with ligands such as tumor necrosis factor α (TNFα), Fas cell surface death receptor ligand (FasL), or IFNγ, and activation of intracellular signaling [134]. These types of cytokines are abundantly secreted by cytotoxic T lymphocytes (CTLs), which orchestrate an adaptive immune response targeting melanocytes in the skin of vitiligo patients [6,155,156,157]. Using an in vivo model of CTL-mediated vitiligo, Jimbo et al. observed reduced coat depigmentation in Fas knockout mice compared to wild-type ones, highlighting the essential role of the axis Fas-FasL as a mediator of melanocyte loss [157]. Investigations have demonstrated that there are abnormal expressions of Fas/FasL and soluble counterparties (sFas/sFasL) in skin lesions and peripheral blood of vitiligo patients [158]. Interestingly, it has been reported that polymorphisms in the Fas promoter regions FAS-1377 AA and FAS-1377 AG have been correlated with increased risk of vitiligo, although such polymorphisms have been associated with diminished promoter activity [159,160].

#### 3.2.2. Necroptosis

Fas activation is also involved in necroptosis, a form of programmed cell death resembling necrosis. Necroptotic cells undergo marked morphological changes due to loss of plasma membrane integrity and release of intracellular contents, making this process highly immunogenic. This characteristic renders this type of cell death highly immunogenic. Necroptosis has been strongly linked to vitiligo, as melanocytes from perilesional skin affected by vitiligo show significant augmented phosphorylated receptor-interacting protein kinase 3 (RIP3) and phosphorylated mixed lineage kinase domain-like protein (MLKL), both key markers of necroptosis [161]. Like apoptosis, in addition to extrinsic triggers, intrinsic factors (mitochondrial-derived ROS) might result in necroptosis. Nonetheless, in the case of apoptosis, cytokine release is minimal, while during necroptosis, this occurrence is markedly evident, leading to vigorous inflammation involving maturation of interleukin IL-1β and nucleotide-binding domain and leucine-rich repeat-containing (NALP) inflammasome multiprotein complex assembly [162]. Differences might consist in membrane rupture occurring during necroptosis that facilitates abundant damage-associated molecular pattern (DAMP) release. By contrast, apoptosis is mostly resolved with phagocytic clearance because caspases regulate the packaging of cellular components into apoptotic bodies prior to cell degradation without the release of cell contents, a mechanism that prevents inflammation [163,164].

#### 3.2.3. Pyroptosis

Another inflammatory form of cell death is pyroptosis. Pyroptosis is a newly discovered modality of programmed cell death that is mediated by pyroptotic caspases. This process triggers rapid rupture of the plasma membrane and initiates the release of pro-inflammatory intracellular contents. As both necroptosis and pyroptosis are lytic forms of cell death, they result in the uncontrolled release of inflammatory mediators such as high-mobility group box 1 (HMGB1), interleukin-33 (IL-33), and S100 alarmin proteins—all of which have been reported as markers associated with vitiligo [165]. Since HMGB1 inhibits the expression of Nrf2 and downstream antioxidant genes in melanocytes, it promotes CXCL16 and IL-8 secretion from keratinocytes and supports dendritic cell maturation, and its release worsens oxidative stress-promoted autoimmunity [166].

#### 3.2.4. Ferroptosis

Dysregulation of iron metabolism and phospholipid peroxidation leads to toxic accumulation of ROS and triggers ferroptosis, an iron-dependent pro-inflammatory cell death modality. GPX4 is considered a key marker of ferroptosis due to its key role in catalyzing lipid peroxide reduction, which prevents the oxidation of membrane lipid components [167]. Several investigations [168] have shown a reduction in GPX levels in the serum and tissues of patients with vitiligo, and genetic studies indicated that GPX1 polymorphism is associated with vitiligo susceptibility [169]. Moreover, unbalanced fatty acid composition is a feature of vitiligo serum composition [170]. Thus, with GPX4 being a selenium-dependent enzyme, supplementation of this element might have applications in vitiligo therapy. Using single-cell RNA-seq datasets, Zhang and collaborators demonstrated that pyroptosis and ferroptosis are involved in melanocyte destruction in vitiligo skin [171]. Detailed gene expression studies have shown that melanocytes from vitiligo patients express lower levels of ferroptosis suppressor genes compared to healthy controls, indicating an increased sensitivity of vitiligo melanocytes to ferroptosis.

#### 3.2.5. Parthanatos

Parthanatos is a form of regulated cell death based on DNA damage and hyperactivated poly (ADP-ribose) polymerase 1 (PARP1) [172]. Excessive intracellular accumulation of cleaved PAR inhibits mitochondrial oxidative respiratory chain enzyme activity, and leads to mitochondrial metabolism disorder, chromatin condensation, and DNA fragmentation, mediating parthanatos [173,174]. Oxidative stress is one of the major factors responsible for PARP1 upmodulation. The involvement of PARP1 in melanocyte cell death has been linked to chemokine-induced stimulation in melanocytes [175]. High PARP1 activity, associated with reduced Sirt1 activity, is a common trait of metabolic diseases [176]. Tulic and collaborators proposed that in IFNγ-primed melanocytes, CXCL10 activating CXCR3B causes downstream cleavage of PARP1, leading to apoptosis [177]. However, the role of parthanatos in vitiligo pathogenesis through PARP1 activation needs to be investigated.

#### 3.2.6. Autophagy-Related Death

Autophagy is a complex catabolic process used by eukaryotic cells during stress conditions to resist energy by macromolecule recycling and to eliminate nonfunctional organelles [178]. Occasionally, in a context-specific way, dysregulated autophagy may turn from a survival mechanism into a cell demise condition [179]. Indeed, blocking autophagy when associated with disrupted mitochondrial transmembrane potential promotes cell death [180]. Autophagy-dependent cell death occurs independently of apoptosis or necrosis and relies on the autophagic machinery. In this case, inhibition of autophagy, genetically or chemically, prevents cell death [181]. On the other hand, reduced detoxifying activities are dangerous because of the possible accumulation in the cytoplasm or the undesired release in the external environment of damaged elements.

Several studies have implicated deregulated autophagy in vitiligo, reporting both overactive and reduced function. Both conditions may contribute to melanocyte loss, as autophagy deficiency impairs energy recovery, inhibits melanocyte proliferation, and leads to premature growth arrest and accumulation of damaged molecules [182,183]. In line with this idea, high ROS amounts in vitiligo can be attributed, at least in part, to insufficient clearance, and failing to address the autophagy requirement may precipitate cell death [180]. Consistently, autophagy-related 7 (Atg7)-deficient mice present reduced melanin production, intense oxidative stress, and senescent melanocytes, resembling vitiligo melanocytes [182]. On the other hand, considering the interconnection of the autophagic pathway with multiple important intracellular signaling pathways, its chronic stimulation might compromise the overall intracellular network equilibrium. Adenosine monophosphate-activated protein kinase (AMPK) is an energy sensor that positively regulates autophagy in the case of a high AMP/ATP ratio. In contrast, the mechanistic target of rapamycin (mTOR) signaling pathway negatively regulates autophagy by switching from catabolism to growth-promoting anabolism, suppressing autophagy. Due to their antagonistic roles, it is known that AMPK-dependent stimulation of autophagic degradation corresponds to mTOR activity inhibition [184] and the coordination of these important metabolic signaling pathways is relevant [185]. Very recently, our group investigated IGF-1/insulin signaling, observing the coexistence of elevated AMPK, microtubule-associated protein light chain 3 (LC3), and overactivation of mTOR and its target pS6 in vitiligo melanocytes, keratinocytes, and fibroblasts, evidencing the lack of coordination between AMPK and mTOR/S6 [130].

The conversion of LC3I into LC3II, being an indicator of autophagic vacuole closure, is considered a standard marker for autophagosome biogenesis. LC3II has been documented by several in vitro studies of vitiligo [130,186,187]. In vivo, p62 expression, which inversely correlates with autophagic activity, is reduced in lesional skin of both active and stable vitiligo patients compared to healthy controls, while LC3-II and Atg5 levels are elevated. Notably, stable vitiligo lesions show a higher LC3-II/I ratio and lower p62 expression than active lesions. Increased autophagic activity was also observed in perilesional skin of stable but not active vitiligo patients. These findings suggest a protective or restorative role of autophagy in mitigating disease progression [183].

Conversely, autophagy dysfunction in melanocytes impairs their proliferative capacity and disrupts Nrf2 signaling, leading to a compromised antioxidant defense system [179].

Yang and collaborators reported “Autophagy” as the most enriched pathway with a prediction of an inhibitory signature associated, at the protein level, with high p62 expression and a low LC3II/LC3I ratio in vitiligo lesional skin [188]. More recently, using combined bioinformatic mRNA analysis and protein profiles of lesional skin biopsies, Zhao et al. identified 20 upregulated genes, and 24 downregulated genes associated with autophagy in vitiligo [189]. However, in the same study, levels of Atg5 protein expression were augmented while LC3 and p62 proteins were significantly reduced compared to control samples. An additional bioinformatic-based study extended the proof of dysautophagy to vitiligo blood cells, further underlying the systemic nature of the disease [190].

Reduced mitophagy, a selective autophagic process involved in mitochondrial quality control, has been observed in melanocytes derived from vitiligo patients, potentially leading to the accumulation of dysfunctional mitochondria. It has been proposed that in vitiligo, the clearance of damaged mitochondria via mitophagy is impaired due to elevated p53 activity, which is triggered by increased hydrogen peroxide levels. As a result, these defective mitochondria persist in generating ROS, thereby exacerbating oxidative stress and ultimately initiating apoptosis, through either the release of mitochondrial cytochrome c or the recruitment of cytotoxic T cells [187].

Interestingly, a correlation has been reported between the mRNA expression levels of mitophagy-related genes and immune cell infiltration in vitiligo-affected skin [188].

Overall, an enormous quantity of data has proven deregulated cell death in vitiligo melanocytes. However, whether vitiligo melanocytes are more prone to unjustified cell death or whether vitiligo melanocytes are spontaneously highly damaged cells and consequently predisposed to accelerated turnover is not clear. In both cases, the possibility of targeting melanocyte cell death in vitiligo patients is relevant due to the possible release of neoantigens and consequent immune cell engagement.

### 3.3. Targeting Premature Senescence Pathways in Vitiligo

In the pathogenic landscape of vitiligo, the accumulation of senescent cells and the presence of senescence markers are diffuse traits of the epidermis and the dermis [74]. Continuous exposure to oxidative injury is mainly responsible for the induction of senescence in melanocytes from the skin of vitiligo patients [74]. Compared to normal counterparts, vitiligo melanocytes show increased expression of p53 and downstream factors p21 and p16, which explains the reduced proliferative capacity. The senescent phenotype is associated with intense secretory activity [38,74,191]. Vitiligo melanocytes increase the production of IGFBPs, MMPs, IL-6, and cyclooxygenase-2 (Cox-2) [38]. Demonstrating that IFNγ/TNFα accelerates melanocyte cellular senescence, Dong and collaborators suggested that the persistence of these Th1 cytokines in vitiligo skin might undermine repigmentation following treatments [192]. Another study identified in the elevation of ROS as the mechanism responsible for JAK2 activation and high p21 expression when IFNγ-induced melanocyte senescence [193]. In vitro UV-irradiated senescent melanocytes are characterized by melanosome transport dysfunction resulting in intracellular melanosome accumulation [194], which may further exacerbate oxidative stress. In vitiligo skin, melanocytes are embedded in a pro-senescence environment since keratinocytes also show some senescent features. Bondanza et al. showed that vitiligo keratinocytes had a shorter life span with an increased p53 expression level [195], a phenotype in agreement with other studies demonstrating the reduced proliferative potential of vitiligo keratinocytes [196]. In rapidly dividing cells such as epidermal keratinocytes, the impact of senescence may be limited due to the substantial stem cell reservoir. Irreversible cell cycle arrest, characteristic of senescence, typically occurs in fully differentiated keratinocytes. Thus, senescence in epidermal keratinocytes is generally thought to affect mainly the metabolic and paracrine functions of basal and suprabasal layers [197]. The pro-senescent phenotype observed in vitiligo keratinocytes gains biological relevance considering their physiological supportive role within the epidermal melanin unit. Senescent keratinocytes release a variety of bioactive molecules collectively known as SASP factors. The SASP has been identified as a major contributor to tissue dysfunction and chronic low-grade inflammation [198]. In this context, Becatti and colleagues reported hyperphosphorylation of p38 MAPK, activation of NF-κB, increased ROS levels, and abnormal expression of inflammatory cytokines in keratinocytes from perilesional skin [199]. Like melanocytes and keratinocytes, fibroblasts from vitiligo skin exhibit a senescence-like phenotype, resembling that of transdifferentiated myofibroblasts and cancer-associated fibroblasts [200,201,202]. Compared to normal fibroblasts, nonlesional vitiligo fibroblasts exhibit an enlarged cell surface and increased expression of α-smooth muscle actin (α-SMA), which forms prominent cytoplasmic stress fibers. These fibroblasts also display elevated levels of reactive oxygen species (ROS) and enhanced production of pro-inflammatory cytokines. Notably, a conditioned medium from vitiligo fibroblasts has been shown to reduce E-cadherin expression in melanocytes, thereby promoting their detachment from the epidermis [197].

## 4. Alterations of Metabolism in Vitiligo Melanocytes

**Figure 4:**
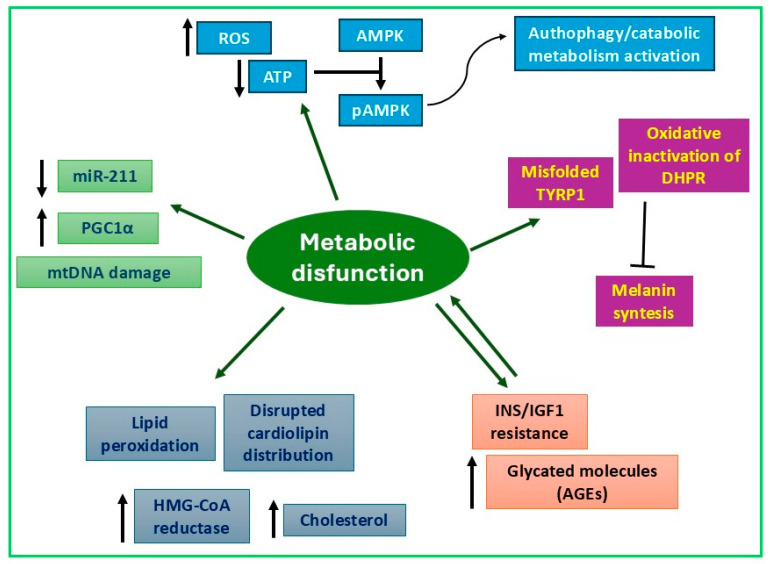
Intrinsic metabolic abnormalities in vitiligo. Metabolic dysfunctions increase melanocyte susceptibility to stress and immune-mediated damage. Metabolic impairment includes defects in glucose and lipid metabolism, and reduced energy availability due to impaired autophagic and catabolic processes, which are further exacerbated by lineage-specific anabolic activities such as melanin synthesis and distribution.

Intracellular metabolism refers to the series of biochemical processes occurring within a cell to maintain its functions and sustain life. This complex network includes interconnected pathways responsible for nutrient breakdown to produce energy, molecule synthesis for cell growth and repair, and the regulation of various metabolic activities. Major intracellular metabolic pathways include glycolysis, the pentose phosphate pathway, and mitochondrial metabolism (oxidative phosphorylation). Intracellular metabolism is tightly regulated to maintain cellular homeostasis and adapt to changing environmental conditions. It also acquires specific characteristics depending on cell type, as different cells exhibit distinct metabolic profiles tailored to their functions and energy requirements. For instance, keratinocytes focus on rapid proliferation and differentiation, utilizing glycolysis for energy to support their role in forming a protective barrier. In contrast, dermal cells, such as fibroblasts, engage in a more complex metabolism, relying on oxidative phosphorylation to generate ATP for collagen and elastin synthesis, which are essential for skin structure and elasticity. Additionally, dermal cells are involved in the metabolism of various growth factors and cytokines, contributing to skin repair and immune responses. This metabolic divergence reflects their distinct functions in maintaining skin health and integrity. Melanocytes primarily rely on aerobic metabolism to produce energy, utilizing glucose and fatty acids to support their functions in melanin and melanosome production and cellular maintenance. Melanocytes also synthesize various antioxidants to combat oxidative stress induced by UV exposure. Additionally, they possess a unique metabolic pathway that converts tyrosine into melanin, a pigment essential for protecting the skin from UV-induced damage. In the pathogenesis of vitiligo, alterations of cell metabolism play a crucial role in determining the “intrinsic defect” underlying melanocyte damage (Figure 4) [6,155,156].

In the epidermis, lipid production stands apart for its amount and heterogeneity due to production for efficient skin barrier formation. Discordant data have been reported concerning stratum corneum hydration in vitiligo-depigmented skin, but decreased hydration and elasticity and an increase in sebum production have been observed in lesions treated with phototherapy compared to untreated depigmented skin [203,204]. From the skin barrier point of view, barrier recovery after tape stripping of the stratum corneum is delayed in vitiligo [203]. In the lesional skin of vitiligo patients, keratinocytes exhibit altered expression of genes corresponding to protein involved in cornification and differentiation, along with marked changes in epidermal thickness and overall tissue architecture [196,205,206]. However, due to the proven relationship between epidermal extracellular lipid content and pH, which in turn largely depends on the phototype, it is not possible to exclude that impairment of skin barrier functionality in vitiligo is secondary to reduction in/loss of melanocytes. By applying a lipidomic approach, Kovacs et al. proved alteration of keratinocyte differentiation with altered lipid composition due to reduced biosynthesis of ceramides associated with defects in cell–cell junctions, impaired energy metabolism, and abundant inflammatory mediators [196]. The centrality of metabolic competence for keratinocyte differentiation emerged by the demonstration that pioglitazone, an antidiabetic drug, partially restores the correct synthesis of ceramides and keratins necessary for an efficient skin barrier [207]. Overall, evidence on the biophysical properties of vitiliginous skin indicates altered epidermal characteristics, highlighting the potential relevance of these changes in the context of therapeutic strategies. Interventions aimed at restoring or enhancing skin barrier composition and function may represent a novel and complementary approach to managing vitiligo and potentially limiting its progression.

Basal intracellular melanocytes’ metabolism is finely regulated, and it is fully integrated in the synthesis of melanin pigments [208,209]. Thus, it is not a surprise that melanocyte-inducing transcription factor (MITF, the master regulator of melanocyte differentiation), in parallel with genes involved in melanin synthesis and melanosome biogenesis (TYR; tyrosinase-related protein 1, TYRP1; tyrosinase-related protein 2, TYRP2; Melan-A (MLANA); premelanosome protein, SILV; solute carrier family 24 member 5, SLC24A5), also regulates proteins affecting mitochondrial biogenesis such as peroxisome proliferator-activated receptor-γ (PPARγ) and PPARG coactivator 1 alpha (PGC1α) [209,210]. Furthermore, RNA sequencing and metabolomic analyses comparing normal melanocytes and PIG3V cells, an immortalized human vitiligo cell line, have identified an interesting mechanism in which the constitutive downregulation of miR-211 in pathological melanocytes allows the upregulation of its target and mitochondrial biogenesis master regulator PGC1α [211]. miR-211 seems to play a unique role in melanocyte biology since it acts as a metabolic switch in nonpigmented melanoma cells [212] and depletion of miRNA-211 promotes vitiligo. Moreover, loss of miRNA-211 expression correlated with the activity of the disease in nonlesional, perilesional, and lesional skin [211]. Moreover, intense pigmentation is reported to correlate directly with the activity of catalase in human melanocytes in vitro and ex vivo [52]. Supporting a positive feedback mechanism, UV-induced PGC1α protein expression stimulates tanning response through the direct induction of MITF expression [213]. Other studies have demonstrated that mitochondrial fission and fusion processes influence melanogenesis (inducing or inhibiting, depending on the specific cellular contexts) through ROS-mediated mechanisms [214,215].

In terms of energetic metabolism, an adequate supply of ATP is of pivotal importance in melanocytes to cope with stress conditions and to fuel melanin biosynthesis. Compared to normal melanocytes, vitiligo melanocytes show reduced ATP production due to defective mitochondrial respiration, which serves as both a cause and a consequence of elevated ROS production. As a compensatory mechanism for energy production, some glycolytic enzymes (hexokinase 2, HK2; pyruvate dehydrogenase kinase 1, PDHK1; pyruvate kinase M2, PKM2) are upmodulated in vitiligo melanocytes [81].

According to reduced ATP levels, vitiligo melanocytes display hyperphosphorylation and activation of the primary kinase involved in nutrient sensing, AMPK [186]. AMPK constitutes an important axis together with the mTOR kinase in regulating global cellular metabolism and cell growth [216]. In accordance with AMPK activation, vitiligo melanocytes show overactive autophagy [186]. However, the catabolic recycling process probably does not compensate for the energetic status, and additional treatments targeting mitochondria activity might be helpful. Interestingly, improved metabolic and energetic status of vitiligo melanocytes has been obtained by stimulating the transcriptional activity of PPARγ, one of the master regulators of mitochondrial biogenesis and function, using pioglitazone, which restores ATP production and mitigates intracellular ROS [217].

Alterations in melanin biosynthesis have been observed in melanocytes from vitiligo patients [1]. Aberrant expression of TYRP1, along with impaired folding in association with calnexin, disrupts the biochemical process of melanin production, leading to the accumulation of toxic melanin intermediates that contribute to melanocyte damage [214].

Moreover, the pro-oxidant environment characteristic of vitiligo has been shown to chemically oxidize the catalytic site of dihydropteridine reductase (DHPR), resulting in its inactivation. In this form, DHPR is unable to regenerate the essential cofactor (6R)-L-erythro-5,6,7,8-tetrahydrobiopterin (6BH4), which is critical for TYR-mediated hydroxylation of L-tyrosine. This leads to impaired melanin synthesis [215].

Finally, a noteworthy crosstalk between aberrant signaling of the pro-inflammatory cytokine TNFα and melanin biosynthesis has been described by Singh et al. Their study demonstrated that TNFα exposure reduces MITF and TYR expression in melanocytes, resulting in decreased melanin content [216].

Collectively, these findings indicate a reduction in lineage-specific melanocyte function, which may predict or contribute to melanocyte loss in vitiligo.

Lipid profiles of vitiligo melanocytes and PBMCs differ from those of healthy controls. Lipid peroxidation and an altered pattern of cardiolipin distribution in the plasma and inner mitochondrial membrane have been observed, and they are associated with an increased level of cholesterol and greater expression of the cholesterol biosynthesis enzyme 3-hydroxy-3methyl-glutaryl-CoenzymeA-reductase (HMG-CoA reductase) [33,218]. Consistently, previous studies reported that lipid-lowering drugs can reverse the depigmentation of vitiligo [219,220].

Keratinocytes isolated from normally pigmented skin of vitiligo patients exhibit altered lipid metabolism, characterized by impaired upregulation of key enzymes involved in the ceramide biosynthetic pathway following calcium-induced differentiation. This defect disrupts the differentiation process and may compromise the integrity of the skin barrier [82].

Several studies have also demonstrated the critical role of the insulin-like growth factor receptor (IGFR) and its downstream signaling pathways in regulating keratinocyte proliferation, differentiation, and survival [221].

Very recently, we provided evidence of Ins/IGF-1 resistance at the cellular level involving melanocytes, keratinocytes, and fibroblasts of vitiligo patients. In vitiligo cells, IGF-1 and insulin stimulation contributes to the aggravation of the energetic imbalance, a condition that promotes excessive glucose import responsible for nonenzymatic overproduction of advanced glycation end products (AGEs). Keratinocytes appeared to be particularly sensitive to glucotoxicity, as evidenced by the marked rise in ROS and pro-inflammatory molecules [130]. As discussed above, autophagy is a lysosome-dependent catabolic process deputed to maintaining cellular nutrient and energy demand balance. Consequently, starvation, including impaired glucose metabolism, which simulates starvation, is a stronger stimulus of autophagy. In line with the worsened ATP availability, vitiligo cells under continuous stimulation of IGFR and INSR strongly activated autophagy [130].

Cellular metabolic impairments described at the cellular level correspond to systemic alterations which resemble the spectrum of metabolic syndrome. Different studies converge in reporting systemic dyslipidemia and hyperglycemia in vitiligo patients [222,223,224]. Compared to healthy individuals, patients affected by vitiligo possess higher blood levels of triglycerides and LDL cholesterol, while HDL cholesterol values are lower than healthy controls. On the other hand, fasting blood glucose levels are significantly higher in vitiligo patients but in the range of subclinical values [224]. The presence in the peripheral blood of elevated quantity of AGEs confirmed a disorder in glucose metabolism [170]. Disordered glucidic metabolism might contribute to melanocyte fragility and subsequent loss in vitiligo.

From the epidemiological point of view, there is increasing evidence that individuals with vitiligo may have a higher prevalence of metabolic syndrome [170,225,226,227]. Patients with active or severe non-segmental vitiligo tend to show a greater frequency of metabolic syndrome [227]. Epidemiological and genetic evidence indicates a significant correlation between diabetes and vitiligo that is not restricted to type 1 but also involves type 2 [226,228].

Overall, these findings reveal profound alteration of multiple different metabolic pathways in vitiligo, contributing to melanocyte loss and underlying a systemic metabolic impairment in patients. Evidence for therapeutic strategies to correct vitiligo by metabolic approaches and existing clinical trials are summarized in Table 1.

## 5. Conclusions

Vitiligo remains challenging to cure. Although cosmetic repigmentation can be achieved, success rates are generally low, and disease flare-ups are common even after clinical stabilization [228]. Furthermore, the presence of subclinical metabolic dysfunctions in clinically unaffected skin underscores the need for preventive or maintenance therapies. Therefore, maintaining repigmentation remains a key goal in vitiligo management, even in the “JAK inhibitor era” [229,230].

In this review, we have discussed the principal mechanisms contributing to vitiligo pathogenesis: (i) oxidative stress, acting as a central hub that initiates and amplifies multiple pathological pathways; (ii) synergic cell death mechanisms, such as apoptosis, necroptosis, and autophagy, collectively driving melanocyte destruction; (iii) senescence, impairing cell function and promoting local immune activation; (iv) metabolic dysfunction, leading to chronic disruption of cellular homeostasis and offering opportunities for targeted therapeutic interventions through molecular profiling.

Advancing our mechanistic understanding of these interconnected processes opens promising avenues for the development of personalized treatment strategies. Such precision medicine approaches may not only halt or reverse disease progression but also improve clinical outcomes by addressing the molecular heterogeneity of vitiligo. Continued research into these core mechanisms is essential to translate basic scientific insights into effective, patient-tailored therapies.

## Figures and Tables

**Table 1:** Legend: Clinical trials were extracted from clinicaltrials.gov and euclinicaltrials.eu, selecting for “active recruiting”, “recruiting”, “active, not recruiting”, “active, not yet recruiting”, “completed”. “Unknown status” means that the study has passed its completion date, and its status has not been verified in more than two years, and “Withdrawn” studies were excluded. Studies directly targeting the immune system and regenerative medicine-based approaches were also not included.

	Pathogenic Mechanism	Experimental Evidence	Proposed Target	Clinical-Level Observational Trials	Clinical-Level Interventional Trials
**Oxidative Stress**	Chronic impairment of redox equilibrium and diminished capacity to activate cellular defense mechanisms after injury	In vivo and in vivo ROS accumulation, reduced activity of antioxidant enzymes, low level of small scavenger molecules	Topical and orally supplemented enzymatic and nonenzymatic antioxidant Nrf-2 activation	Stress response in vitiligo (NCT02797574) Investigating IGF-1 against oxidative stress in vitiligo (NCT05812079)	Oral Ginkgo biloba (NCT00907062, NCT01006421 *) Investigating IGF-1 against oxidative stress in vitiligo (NCT05812079) Apremilast (NCT03036995) Oral GLISODIN–SOD (NCT03941808 *) Total Glucosides of Peony (NCT03608917 *)
**Senescence**	Loss of fully functional melanocytes, possible recruitment of immune system	Presence of senescence markers (p16, p21, GADD45) and SASPs (MMPs, IGFBPs, cytokines)	Proposed use of senolytic/antioxidant repurposed compounds	No data at clinical level, preclinical evidence	Topical Ramamycin (NCT05342519 #)
**Melanocyte Cell Death**	High melanocyte turnover, likely release of DAMPs and amplification of immune response	Presence of apoptosis-, necroptosis-, ferroptosis-, pyroptosis-, and autophagy-related cell death markers	Blockage of intrinsic and extrinsic processes implicated in cell death	Evaluation of pyroptosis (NCT06261086) Histochemical study of apoptosis markers (NCT05869942)	Oral Afamelanotide (NCT06109649 *) Subcutaneous Afamelanotide (NCT05210582, NCT04525157 *, NCT01430195 *, NCT01382589 *)
**Metabolism**	Abnormal lipid profile, dysfunctional mitochondria, accumulation of glycated molecules	In vitro and in vivo increased cholesterol, lipid peroxidation, AGEs Systemic dyslipidemia and hyperglycemia	mTOR pathway inhibition, correction of lipid profile, inhibition of HMG-CoA reductase	Metabolic syndrome comorbidities (NCT03622320) Glucose and lipid metabolism (NCT05968235) Association with other autoimmune diseases (NCT04789993) Circulating mitochondria (NCT05525741)	Oral Sinvastatin (NCT01517893), Oral Torvastatin (NCT02432534), Oral Atorvastatin (NCT03247400, NCT02432534) Oral vitamin D (NCT04872257 *, NCT05364567 *) Topical Ramamycin (NCT05342519 #)

* Treatment associated with phototherapy; # same study possibly targeting more pathogenic mechanisms.
